# Does Mirror Imaging a Radiograph Affect Reliability of Age Assessment Using the Greulich and Pyle Atlas?*

**DOI:** 10.1111/j.1556-4029.2012.02150.x

**Published:** 2012-04-17

**Authors:** Lucina Hackman, Sue Black

**Affiliations:** 1 Centre for Anatomy and Human Identification, University of DundeeMSI/WTB/JBC Complex, Dow Street, Dundee DD1 5EH, U.K.

**Keywords:** forensic science, forensic anthropology, age estimation, Greulich and Pyle, radiographs, age assessment living

## Abstract

Age estimation is routinely undertaken by comparing radiographs of the individual in question to published reference samples of individuals of known age. This study examines the reliability of age estimation utilizing the Greulich and Pyle atlas in relation to both left- and right-hand/wrist radiographs and explores whether reversing right-hand/wrist radiographs, so that they are in the same anatomical orientation as those images used in the atlas affects reliability. A total of 403 left-hand/wrist radiographs and 415 right-hand/wrist radiographs were age assessed using the Greulich and Pyle atlas. Analysis showed that there is no significant loss in reliability when radiographs of the right hand (women *R*^2^ = 0.887 and men *R*^2^ = 0.907) are utilized instead of the left (women *R*^2^ = 0.939 and men *R*^2^ = 0.940) or when they are assessed as mirror images to those printed in the reference atlas (reversed female left hand *R*^2^ = 0.929 and reversed male left hand *R*^2^ = 0.931).

The estimation of age from the living utilizes atlases and other published data sources to compare the degree of hard tissue maturation reached by the individual in question with individuals of known age. However, these atlases were not devised with forensic evaluation in mind but were developed to assess clinical growth and maturation in the child. Therefore, the forensic community is utilizing an approach designed for a very different purpose: to assign an age to a person who either cannot, or chooses not to, reveal their true chronological age. It is essential therefore that the methodologies are tested fully and robustly, to ensure that they are fit for purpose and meet current and future issues of legal admissibility.

A trio of images are recommended in age estimations and comprise a radiograph of the left hand/wrist, an orthopantomogram, and if possible feasible computed tomography scans of the medial clavicles (1). It is argued that a combination of these images will give an age estimation that is appropriate for use in judicial circumstances (2). For each of the three images, the estimation of chronological age is achieved by comparing the image of the relevant anatomical area in the subject, to a published reference standard. One of the most commonly used standards for the hand/wrist region is the atlas of Greulich and Pyle (3) despite it utilizing images of children from over 70 years ago.

Greulich and Pyle (3) justified the use of the left-hand/wrist images in their atlas by following guidelines laid down by “The International Agreement for the Unification of Anthropometric Measurements to be Made on the Living Subject” (4). These guidelines stated, within a list of general principles on anthropometric measurement, that “For ‘paired’ measurements, the left side is recommended” (4, p. 62). Greulich and Pyle (3) also argued that the left hand is less likely to suffer injury or trauma because, within any given population, the number of individuals who are right handed is larger than the numbers who are left handed.

The question originally raised by the exclusive use of the left side of the body in the atlases was whether this is reflective of the maturational status of the right side within the same individual. Greulich and Pyle (3) addressed this question by referencing the work of Dreizen et al. (5) who examined the relationship between maturational levels of the right and left hands of over 400 children. They found that while differences did exist between the two sides of the body, these were so minor that they were insignificant in relation to the estimation of maturational stages of the skeleton as a whole and this result was subsequently supported by other studies (6).

In addition to the issues of methodological robusticity, there are a number of justifications as to why the accuracy of right-side hand/wrist radiographs in age estimations should be examined further. In the United Kingdom, X-rays are not taken for the purpose of age estimation without the informed consent of the individual (7). For those individuals who have been in the country for a period of time, it may be possible to trace and access radiographic images taken during treatment at an Emergency Department should permission for radiography not be granted. These may not be of the “ideal” left side of the body, especially because it is more likely that the right hand is imaged as the result of potential injury (8, 9). Also radiographs may have been ordered by the Court prior to consultation for advice, and the right side of the body may have been imaged. A return to the individual for a left-hand radiograph may not be possible and does not reflect good ethical practice. Further, it is possible that trauma or untreated developmental disorders might render the use of the left side of the body unsuitable for analysis.

Today many age estimations can, and do, become the focus of court proceedings, and so there is a strong argument for the need to demonstrate that age assessments using the right side of the body carry similar discriminatory value to those undertaken utilizing the left side as per the traditional recommendations. As the suggested methodology for age estimation in the living recommends the use of the left hand/wrist, it is vital that any practitioner understands the implications for alterations to this ideal requirement and how that might impact upon their reliability and accuracy. Finally, proving that the right and left sides of the body are interchangeable for the purposes of age estimation would permit data to be combined for the purposes of research, increasing the data pool available for analysis as it is, quite rightly, no longer possible or permissible for health reasons to obtain longitudinal radiographic data. Many of the resource data available dates from more than half a century ago and with alterations to nutritional status, environmental influences, and other factors that will impact on secular trend (10–12), it is essential that methods are continually updated by testing on modern samples of different origin.

This study therefore set out to answer two questions: first, is the reliability of age estimation undertaken using the Greulich and Pyle approach comparable for right-hand radiographs; and second, would mirroring the image of the right hand, so that it is in the same anatomical orientation as the images in the atlas (i.e., left), cause a significant change in reliability when undertaking an age estimation?

## Materials and Methods

Radiographic images were examined for 818 individuals (545 men and 273 women) between the ages of 1 and 21 years (Table 1). The images were sourced from Ninewells Hospital in Dundee, which is a large NHS teaching facility that serves a community of approximately 400,000 across the Tayside area in the northeast of Scotland. The life expectancy in this area of Scotland is 78.8 years (women 80.6 years and men 76.9 years), which is slightly higher than the national average (13). The catchment area across Tayside has a population which includes 17% living in poverty as described by the Scottish Indices of Multiple Deprivation, 20% are students because of the presence of the local universities, and approximately 1.9% are considered to be nonwhite, although a large dependence on agriculture in the surrounding area means that this varies by season with an increase in transient migrant workers during some months of the year (14). It was not possible to know and therefore record the ethnicity or socioeconomic background of each individual as the clinical records did not require this to be noted. The images had all been taken as part of medical diagnosis and/or treatment of children and adolescents who attended the Accident and Emergency Department of the hospital.

**TABLE 1:** Number of radiographic images separated by sex and side.

Sex	Side	Number of Images
Female	Left hand/wrist	156
Female	Right hand/wrist	117
Male	Left hand/wrist	247
Male	Right hand/wrist	298
		818

A record was made of the sex, date of birth, date of image acquisition, and side of the body depicted in the radiograph. The age of the individual was calculated as the difference between the date of birth and the date of the radiograph. Images that contained recent untreated fractures were used, but if treatment had commenced or there had been a previous fracture of that joint, then these images were not included. Individuals who had been diagnosed with a clinical disorder such as precocious puberty, osteogenesis imperfecta, or who had experienced extensive medical intervention for illnesses such as cancer were also not included in an attempt to keep the sample as clinically “normal” as possible. It should be noted that the images examined were taken for diagnostic or therapeutic purposes only, and therefore, the radiographer imaged the area of the hand/wrist that was relevant for their purposes and in an orientation that met the needs of the task. This resulted in a number of images that could not be utilized because of poor contrast or unsuitable anatomical orientation for the purposes of comparison with the atlas. It should also be realized that for each individual, it was normal for only a right or a left hand to be radiographed and few individuals were represented by both hands; therefore, bilateral symmetry within an individual could not be examined.

Table 2 indicates the number of radiographic images available for each age cohort grouped into calendar years. The lower number of individuals in the very young age groups is to be expected as they are less prone to requiring emergency orthopedic attention. Older age groups contain larger numbers of individuals reflecting greater exposure to higher risk occupations including physical activities and sports, which may result in attendance at an Accident and Emergency Department following an accident.

**TABLE 2:** Number of radiographic images separated by sex, side, and age.

Years	Female Left	Female Right	Male Left	Male Right	Total
1–2	3	0	3	1	7
2–3	3	1	3	1	8
3–4	3	3	3	2	11
4–5	6	2	6	2	16
5–6	1	1	7	2	11
6–7	7	0	2	7	16
7–8	8	6	8	6	28
8–9	3	3	8	7	21
9–10	10	5	12	9	36
10–11	18	8	15	8	49
11–12	6	6	17	13	42
12–13	10	8	15	33	66
13–14	17	10	16	25	68
14–15	10	11	18	27	66
15–16	5	11	19	23	58
16–17	10	11	19	15	55
17–18	8	7	21	23	59
18–19	12	8	19	33	72
19–20	6	5	19	29	59
20–21	10	11	17	32	70
	156	117	247	298	818

Skeletal age estimation was undertaken for each of the radiographs, by the first author, using the Greulich and Pyle atlas (3). This assessment was undertaken without prior knowledge of the chronological age of each of the children examined. Owing to well-recorded differences in the development of women and men, age estimation was undertaken separately for each sex.

**FIG. 1:**
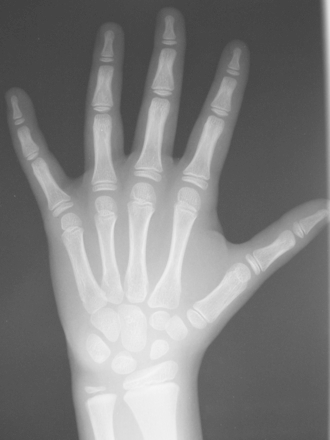
Left-hand/wrist radiograph.

**FIG. 2:**
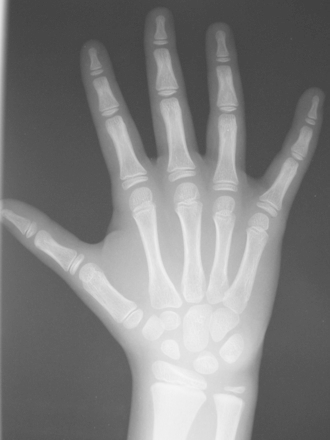
Mirrored left-hand/wrist radiograph.

Upon completion of age assessment for each group, all images (both right and left hands) were rotated across the vertical axis; thus, the left-hand images were reversed, so that they were in the same orientation as a radiograph of a right hand/wrist and the right images were reversed to mimic a left-hand image, demonstrated in Figs 1 and 2. Once the image was reversed, they were again age assessed using the Greulich and Pyle atlas (3), with a delay of 2 weeks between assessments.

An inter-observer test was devised in which 57 randomly selected images from the female left-hand group were age assessed by a second forensic anthropologist with experience of viewing radiographs and a knowledge of, but little experience with, the Greulich and Pyle (3) age estimation system. Any indicator of side on the radiograph such as the large “L” marker used by radiographers was obscured, and the observer was provided with a copy of the Greulich and Pyle atlas but was given no instructions in its use. Two weeks after completion of this test, the original images were reversed across the vertical axis, so that they appeared to be images of the right hand. These images were given to the same observer who was again asked to age assess them using the same atlas. The observer was informed that the images were from female subjects but given no further information. At no point was the second observer informed that they were the same images that had previously been assessed. The images were in digital format, and posttest questioning confirmed that the observer had not made any effort to rotate them during the age estimation process.

Once skeletal age had been assessed, linear regression analyses and correlations were performed for each group and for both observers. All of the radiographs were from different patients as this is a cross-sectional data source, and so it was not possible to compare bilateral reliability of age estimation. A comparison of the regression slopes for each sex and side was compared using Graphpad (GraphPad Software, Inc., La Jolla, CA).

## Results

The 1959 edition of the Greulich and Pyle atlas has separate standards for men and women: in men, the image at which full skeletal maturity has been achieved is “*Male standard 31*” that is assigned a chronological age of 19 years. For women, the corresponding image is that of “*Female Standard 27*” that is assigned a chronological age of 18 years. In this study, all of the radiographs were age assessed up to, and including, those in the 20–21-year age group to confirm when age-related maturation could no longer be identified in the current sample. Within the 18–21-year age groups for women, there were 14 individuals who had not reached the stage of maturity seen in “*Female Standard 27*,” and in the 19–21-year age groups for men, there were 11 individuals who had not reached “*Male Standard 31*,” despite the individual having passed the identified chronological age for these standards (Table 3).

**TABLE 3:** Number of radiographic images where fusion still active after the age at which Greulich and Pyle indicated that it had ceased.

Sex and Side	Number of Images in Age Groupings 18–21 Years for Females and 19–21 Years for Males	Number in Which Fusion Still Active	% in Which Fusion Still Active
Female left hand/wrist	24	7	29
Female right hand/wrist	28	7	25
Male left hand/wrist	61	2	3
Male right hand/wrist	36	9	25

Finding individuals who were still undergoing fusion was not unexpected because in any population, there will be individuals who, for a variety of reasons, achieve maturational milestones at a different chronological age than others (15, 16). The radiographs in the Greulich and Pyle atlas represent the average or median skeletal development for that chronological age and do not illustrate outliers. As these outliers were shown to exist in this cohort, all images were included in the statistical assessments as this is a true representation of the sample.

Linear regression analysis and Spearman’s correlation analysis were undertaken on the data with chronological age treated as the independent variable in all of the equations. Tables 4 and 5 show the results of the analysis of the groups both before and after vertical axis mirroring for each of the observers. Table 4 shows that the regression coefficients remained high for all of the groups, indicating that there is a strong relationship between chronological age and assessed age using the Greulich and Pyle atlas for both sexes and for both sides of the body. The *p*-values for all of the analyses were highly significant (*p* ≤ 0.0001). For three of the four comparisons, males had a marginally higher correlation value than females for the same hand, but this was not statistically significant. In their correct anatomical orientation, there was a slightly higher *R*^2^ value for the left hands than for the right hands. The correlations remained consistently high after mirroring of images for both observers, although interestingly there was a slightly higher *R*^2^ value for the right hands that were reversed to look like left hands. This was true for both males and females. The inter-observer test showed an equal strength of relationship between the correct sided hands and those that were reversed when correlated with chronological age (Table 5).

**TABLE 4:** R^2^-values and regression coefficients by sex and side for the assessments undertaken by the first observer.

	Regression Coefficient	*R*^2^-Value	*p*-Value
Female left hand/wrist	0.894	0.939	<0.0001
Female right hand/wrist	0.859	0.887	<0.0001
Male left hand/wrist	0.979	0.940	<0.0001
Male right hand/wrist	0.940	0.907	<0.0001
Reversed female left hand/wrist	0.879	0.929	<0.0001
Reversed female right hand/wrist	0.893	0.935	<0.0001
Reversed male left hand/wrist	0.963	0.931	<0.0001
Reversed male right hand/wrist	0.957	0.942	<0.0001

**TABLE 5:** R^2^-values and regression coefficients for second observer.

	Regression Coefficient	*R*^2^-Value	*p*-Value
Female left hand/wrist	0.921	0.927	<0.0001
Reversed female left hand/wrist	0.927	0.927	<0.0001

The regression coefficients were compared for each group, before and after rotation, to determine whether the repeatability of age estimation differed significantly as a result of changing the image orientation. Tables 6 and 7 present the results of these comparisons. The results show that regardless of the orientation of the images, the repeatability of the age estimation performed with the Greulich and Pyle atlas did not differ significantly. There were no significant differences between either the slopes or intercepts for any of the groups when “before” and “after” rotation analyses were compared. As a result, pooled regression coefficients can be presented (Tables 6 and 7). The comparison of regression coefficients for the second observer gave a comparable result, indicating that their age estimations for the images in both orientations did not differ significantly (Table 7).

**TABLE 6:** Comparison of regression coefficients between the groups for the first observer.

Sex and Side	Pooled Regression Coefficient	Significance
Female left hand/wrist compared with female right hand/wrist	0.880	NSD
Male left hand/wrist compared with male right hand/wrist	0.960	NSD
Female left hand/wrist compared with female left hand/wrist reversed	0.880	NSD
Female right hand/wrist compared with female right hand/wrist reversed	0.872	NSD
Male left hand/wrist compared with male left hand/wrist reversed	0.971	NSD
Male right hand/wrist compared with male right hand/wrist reversed	0.944	NSD

NSD, no significant difference.

**TABLE 7:** Comparison of regression coefficients of female left hand/wrist and rotated images of female left hand/wrist for second observer.

Sex and Side	Pooled Regression Coefficient	Significance
Female left hand/wrist vs. female left hand/wrist reversed	0.925	NSD

NSD, no significant difference.

There is a requirement on the forensic scientist to ensure that the methodologies they apply to a case are founded on sound principles, are tried and tested on relevant material, and have been published and accepted by peer review. For this reason, many of the approaches that have been faithfully followed in the past need now to be re-examined in a new light leaving no aspect of its validity left unaddressed. The authors of this paper have had cause to present evidence to the courts for the purposes of age estimation in the living (17) and have been confronted with images that do not conform to the textbook requirements of a standard antero-posterior view of the left hand and wrist. It is therefore essential that we interrogate the robusticity of our methodologies to determine whether unanticipated presentation may have a significant effect on the reliability of the results achieved.

The Greulich and Pyle atlas was developed to demonstrate the median point of skeletal maturation at an identified chronological age, despite this limitation, this communication has supported other studies which have shown a strong correlation between age estimated by the Greulich and Pyle atlas and the chronological ages of juveniles from a modern population (18–21). In addition, the results have shown that there is no significant difference in whether a right or a left hand is used for comparison with the reference atlas or indeed whether a radiograph is mirrored across the vertical axis. However, the results did indicate that the relationship is marginally stronger when mirror image matching is not employed (i.e., comparing a right-hand to a left-hand standard), and this is perhaps to be expected given the spatial cognitive skills required in such processes (22). Therefore, although there is no significant difference in the strength of the relationship between chronological age and the selected Greulich and Pyle standard, it is advised that, where possible, left hands should be selected for comparison, but where this is not possible, then images of the right-hand radiograph should be flipped across the vertical axis to maintain a conformity of approach as a standard operating procedure.
